# Compliance with the sepsis resuscitation bundle in patients with severe sepsis and septic shock admitted to Scottish ICUs

**DOI:** 10.1186/cc10661

**Published:** 2012-03-20

**Authors:** JA Davidson, K Dunne

**Affiliations:** 1 Victoria Infi rmary, Glasgow, UK; 2 Forth Valley Royal Hospital, Larbert, UK

## Introduction

Severe sepsis is the second leading cause for admission to critical care and in spite of advanced care remains associated with a high mortality. When implemented the sepsis resuscitation bundle has been associated with a 20% reduction in mortality and is therefore recommended as standard care for all patients with severe sepsis [1].

## Methods

All new admissions to seven west of Scotland ICUs were screened during a 12-week period for evidence of severe sepsis or septic shock. Those meeting the criteria were then assessed for sepsis bundle compliance. The Institute for Healthcare Improvement sepsis resuscitation bundle was taken as standard of care. This has a 6-hour time frame and includes measurement of serum lactate, blood cultures taken prior to antibiotics, antibiotics administered within 3 hours, fluids of 20 ml/kg if hypotensive or hyperlatataemia and use of early goal-directed therapy in the event of persistent hypotension/hyperlatataemia in spite of fluid resuscitation.

## Results

Of the 652 patients screened, 115 met the definition of severe sepsis or septic shock (17.6%). We collected full data from 108 patients, of which 69 patients (63.8%) had severe sepsis and 39 patients (36.1%) had septic shock. Full bundle compliance was 5.6%. Early ICU admission (within 6 hours) was associated with improved compliance with measured lactate (87.3% vs. 60.4%, *P *< 0.01), and where indicated, vasopressor use (94.4% vs. 61.3%, *P *< 0.01), CVP measurement (77.5% vs. 44.4%, *P *< 0.01), and ScvO_2 _measurement (25.6% vs. 2.8%, *P *< 0.01). ICU mortality was 12/64 patients (18.8%) with severe sepsis and 18/38 patients (47.4%) for those with septic shock. Full bundle compliance and mortality was not different for those reaching ICU early compared with those who were admitted after 6 hours.

## Conclusion

At present the sepsis resuscitation bundle is not uniformly implemented. Although compliance with early goal-directed therapy and lactate measurement is better in those reaching ICU early, there is still a large proportion of patients not receiving aspects of the bundle in spite of being in a critical care environment.
